# Neurological outcomes after surgery and postoperative rehabilitation for cervical radiculopathy due to disc disease: a 2-year-follow-up of a randomized clinical trial

**DOI:** 10.1038/s41598-023-31005-z

**Published:** 2023-03-07

**Authors:** Anneli Peolsson, Håkan Löfgren, Åsa Dedering, Mattias Kristedal, Birgitta Öberg, Peter Zsigmond, Johanna Wibault

**Affiliations:** 1 Department of Health, Medicine and Caring Sciences, Unit of Physiotherapy, Linköping University, Linköping, Sweden; 2 Department of Health, Medicine and Caring Sciences, Unit of Clinical Medicine, Occupational and Environmental Medicine Center, Linköping University, Linköping, Sweden; 3 Neuro-Orthopedic Center, Jönköping, Region Jönköping County, and Department of Clinical and Experimental Medicine, Linköping University, Linköping, Sweden; 4 Division of Physiotherapy, Department of Neurobiology, Care Sciences and Society, Karolinska Institutet, Stockholm, Sweden; 5 Department of Activity and Health, and Department of Health, Medicine and Caring Sciences, Linköping University, Linköping, Sweden; 6 Department of Neurosurgery, Linköping University Hospital, and Department of Clinical and Experimental Medicine, Linköping University, Linköping, Sweden

**Keywords:** Neurology, Signs and symptoms

## Abstract

Reports on neurological outcomes in patients with cervical radiculopathy (CR) undergoing surgery and postoperative rehabilitation are important to inform prognosis. This 2-year-follow-up of a randomized clinical trial aimed to compare secondary neurological outcomes between structured postoperative rehabilitation and a standard approach after surgery for CR. A secondary aim was to increase knowledge about recovery of neurological impairments in relation to patient-reported neck disability. Neurological outcomes included assessment of sensibility, motor function, arm reflexes and the Spurling test. A total of 153 and 135 participants (> 70% response rate) completed the clinical examination. Between-group differences, changes over time, and associations between persistent neurological impairments and the Neck Disability Index were investigated. No between-group differences were reported (p > 0.07), and neurological impairments in sensibility, motor function, and a positive Spurling test decreased over time in both groups (p < 0.04). Persistent impairments in sensibility and reflex arm were most frequent at follow-up, whereas, a persistent positive Spurling test, and impairments in motor function were associated with higher NDI score. Neurological outcomes improved over time in patients undergoing surgery for CR with no between-group differences., However, persistent neurological impairments were common, and associated with poorer outcome for patient-reported neck disability.

**Clinical registration**: clinicaltrial.gov NCT01547611, 08/03/2012, Title: Outcome of physiotherapy after surgery for cervical disc disease: a prospective multi-centre trial.

## Introduction

The incidence rate of cervical radiculopathy due to cervical disc disease (CR) has been reported as 83.2 per 100,000 in a general population^1,2^. Patients with CR present with complex symptomatology and report—in addition to neck and arm pain—physical and psychological disability and low health-related quality of life^3,4^. Evidence-based clinical guidelines for the management of CR are lacking, and non-surgical treatments are considered initially given a rather favourable natural course of the condition^5^. Decompression of the nerve root is the established surgical treatment for reducing pain and neurological symptoms in patients with persistent CR, with an overall success rate of 80% on the self-rated Odom scale (excellent to poor)^3,6^. However, remaining pain, neck-specific disability and reduced health-related quality of life are common^3,4,7^. Evidence-based rehabilitation programmes have been suggested to improve patients’ physical function and health after a prolonged period of pain and reduced physical activity preceding cervical spine surgery. Neck-specific exercises are the single most evidence-based treatment in other types of neck pain disorders^8–10^, and are reported to improve neurological function in individuals with moderate/severe chronic whiplash associated disorders (WAD)^9,10^. Neck-specific exercises have been shown to be tolerated postoperatively by patients with CR without any harm^11–13^, but have not been investigated to any significant extent.

Evaluation of neurological outcomes is a recognized part of the clinical examination in individuals with CR. This involves investigating sensibility (pin prick and light touch), motor function and reflexes, and plays an important role in decision-making for surgery^14,15^, but has seldom been investigated in research studies. Existing studies are mostly retrospective evaluations or, in a few cases, smaller prospective studies of self-rated symptoms without a clinical examination at follow-up and often in a mixed population^16–20^. Only two prospective randomized controlled studies (RCTs) included a clinical examination of neurological outcomes and reported improvement over time^21,22^, with overall success rates for disc arthroplasty (> 80%) compared with anterior cervical decompression and fusion (ACDF). Burkus et al.^22^ evaluated motor function, sensibility and reflexes in patients with CR without an overall pooled grading of neurological outcomes. Normal sensory function at baseline was important for overall success after ACDF, but was not related to patient-reported neck-specific function evaluated with the Neck Disability Index (NDI)^23^. To our knowledge, there is no previous study reporting neurological outcomes in patients with CR after surgery and postoperative rehabilitation including neck-specific exercises. Knowledge about neurological outcomes following surgery and postoperative rehabilitation is important to inform patient expectations regarding prognosis. The aim of this study was to investigate secondary neurological outcomes in the first prospective randomized clinical trial of postoperative rehabilitation in patients with CR undergoing surgery. The additional benefits of a structured postoperative rehabilitation programme (SPT) including neck-specific exercise combined with behavioural therapy were compared with a pragmatic standard postoperative care approach (SA). Between-group differences and changes over time in neurological outcomes were considered. A secondary aim was to increase knowledge about recovery of neurological impairments in patients with CR undergoing surgery and postoperative rehabilitation in relation to patient-reported neck disability.

## Methods

### Study design

This is a report of long-term secondary neurological outcomes in a multi-centre RCT of postoperative rehabilitation after surgery for CR (ClinicalTrials.gov NCT01547611, 08/03/2012), performed according to a published study protocol^24^. The study was approved by the regional ethics committee in Linköping, Sweden (M126-08), and was conducted in accordance with the Declaration of Helsinki.

### Randomization

After giving their informed consent, participants were prior to surgery randomized to structured postoperative rehabilitation (SPT), which combined neck-specific exercise with a behavioural approach, or a standard approach (SA), in which patients were not referred to a physiotherapist after surgery^24^. A computerized randomization list created by a statistician (before the study started) was used and administered by the main project leader, who was not involved in the intervention and follow-up. The investigators were blinded to group randomization and were not involved in either surgery or rehabilitation.

### Participants

**Figure 1:**
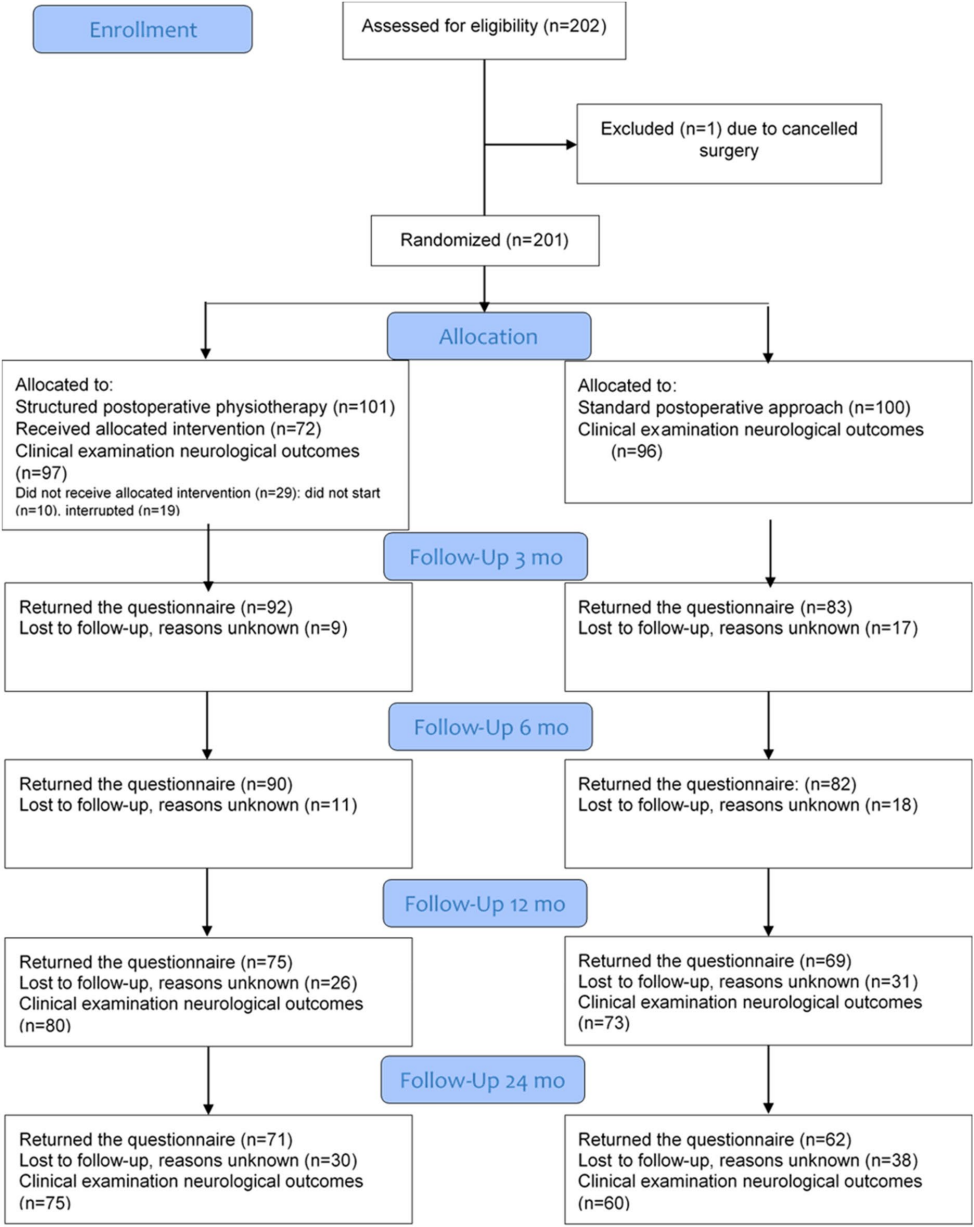
Flow chart of participants included in the analyses of secondary neurological outcomes.

Two-hundred and two participants were consecutively recruited at four neurosurgery/neuro-orthopaedic clinics in Sweden between 2009 and 2012^12,13^, of which 201 underwent surgery (mean age 50; SD 8.4 years, 52% men, neck pain median duration 14 months; arm pain median duration 12 months; IQR 16). The inclusion criteria were: age 18–70 years, persistent radiculopathy symptoms for at least two months, clinical findings of nerve root compression based on examination by a neurosurgeon/neuro-orthopaedic surgeon and compatible with verified cervical disc disease determined by magnetic resonance imaging, and undergoing surgery for CR by either anterior approach (ACDF) or posterior approach with foraminotomy/laminectomy at one to three segmental levels. The exclusion criteria were: myelopathy, previous fracture or luxation of the cervical column, malignancy or spinal tumour, spinal infection, previous surgery in the cervical column, systematic disease or trauma that contraindicated either the rehabilitation programme or the measurements, diagnosis of a severe psychiatric disorder (such as schizophrenia or psychosis), known drug abuse and lack of familiarity with the Swedish language (unable to understand and answer the questionnaires). Of the 201 participants who underwent surgery, 163 were operated on with ACDF using standard cages (i.e. filled with bone substitute or autologous bone collected during decompression; no iliac crest graft was taken) at the clinic where the participant was included. In most cases of multilevel surgery, an anterior plate was added to achieve primary stability. Thirty-eight patients underwent posterior foraminotomy, with or without laminectomy (without fusion). Eight participants did not fulfil the clinical neurological examination at baseline and were excluded from this secondary analysis of outcomes. Thus, the present cohort consisted of 193 participants (Table 1). A total of 153 (79% response rate) and 135 (70% response rate) participants completed the clinical examination at one- and two-year follow-up (Fig. 1). Of the participants attending one-year follow-up, 83 (46%) were men and mean age was 50 (SD 8.2). At the two-year follow-up, 72 (53%) were men and mean age was 50 (SD 8.3). There was no difference in background variables or preoperative neurological outcomes between the patients who attended the neurological clinical examination at follow-up and those who were lost to follow-up (p > 0.194). Patients attending clinical examination at follow-up scored NDI mean value 21 (SD 16.7) at one-year follow-up, and 23 (SD 18.3) at two-year follow-up. There was also no difference in background variables or neurological outcomes at baseline between participants randomized to SPT or SA (p > 0.08) (Table 1).

**Table 1:** Background variables for participants with cervical radiculopathy who underwent surgery and postoperative rehabilitation and were included in the secondary analysis of postoperative neurological outcomes.

	N	Total	SPT (N = 97)	SA (N = 96)
Age, mean (SD)	193	50 (8.4)	50 (8.3)	50 (8.6)
Sex male, n (%)	193	100 (52)	48 (50)	52 (54)
Anterior surgery, n (%)	193	155 (80)	73 (75)	82 (85)
NDI %, mean (SD)	184	43 (14.9)	42 (14.5)	44 (15.4)
Neck pain mm VAS, mean (SD)	188	56 (24.3)	55 (24.9)	57 (23.8)
Arm pain mm VAS, mean (SD)	185	50 (28.0)	52 (26.5)	48 (29.5)
Neurological impairment prick touch, n (%)	193	154 (80)	78 (80)	76 (80)
Neurological impairment light touch, n (%)	193	138 (72)	70 (72)	68 (71)
Neurological impairment motor function, n (%)	191	150 (79)	80 (83)	70 (74)
Neurological impairment arm reflex, n (%)	186	109 (59)	50 (53)	59 (64)
Positive Spurling test, n (%)	142	91 (64)	49 (67)	42 (61)

Results are presented with mean value and standard deviation (SD) or number (n) and percentage (%).

### Postoperative care and rehabilitation

Participants in both groups received the same initial postoperative care at the surgical clinic during the first six weeks, which consisted of information, advice and mobility exercises for the shoulders. After six weeks, participants were instructed to perform mobility exercises for the neck. Neither group used a cervical collar. Participants in the SPT group visited the physiotherapist (referral from the project team) once weekly beginning with postoperative week six, and then twice weekly from postoperative weeks 12 to 24. Participants also performed exercises at home. SPT focused on facilitation and endurance of neck muscles, strengthening of scapular muscles, postural control and increasing overall level of physical activity. The exercises were individually adjusted and progressed for each patient by the treating physiotherapist and registered in an exercise diary. A cognitive behavioural approach consisting of different lessons aimed at improving pain management, coping strategies, ergonomics and self-efficacy was included in the rehabilitation programme. Participants in the SA group received the usual postoperative care after surgery for CR in Sweden. They were advised to seek pragmatic postoperative physiotherapy independently (61%) beginning in postoperative week six if they felt this was necessary, without the need for a referral.

### Outcome measures

Primary outcome measure in the study was patient-reported neck disability evaluated with the NDI (0% = no disability, 100% = major disability)^25^, and the results have previously been reported elsewhere^12^. Report of secondary neurological outcomes in the present study was based on a neurological examination performed before surgery and at one- and two-year follow-up. Neurological examination included a bilateral assessment of sensibility with a pin prick and a light touch in dermatomes C4–C8, motor function assessment with manual muscle testing of the C4–C8 myotomes, arm reflex testing for C5, C6 and C7 with a standard reflex hammer^26^, and Spurling test of provocation of current radiculopathy^27^. Responses were classified as normal or abnormal, including hypoesthesia, hyperesthesia or dysesthesia, or allodynia for sensibility; decreased strength according to the modified Janda scale for motor function (full range of motion was not used); and hyporeflexia or hyperreflexia for arm reflexes^28^. Any abnormal response or asymmetry in at least one of the dermatomes, myotomes or reflexes was classified as impairment in sensibility (prick touch or light touch), motor function or arm reflexes. Participants who presented neurological impairment in sensibility, motor function, arm reflexes or Spurling test preoperatively but no longer at the time of follow-up were classified as recovered for the specific neurological function.

### Statistical analysis

Descriptive statistics are presented with mean value (mean) and standard deviation (SD), or number (n) with percentage (%). Between-group difference in postoperative neurological outcomes including persistent neurological impairments and recovery at one- and two-year follow-up was analysed with the Chi-square test. Change over time in the presence of neurological impairments was analysed in both groups with the Cochrane Q test, and post-hoc tests with the McNemar test. Univariate associations between one-year persistent neurological impairments in sensibility, motor function, arm reflexes and a positive Spurling test were reported. Further, the contribution of the preoperative factors sex, age, surgical procedure, randomization group and preoperative neurological impairments to one-year persistent neurological impairments were investigated with multiple forward logistic regression analysis. The results are presented with odds ratio (OR) and 95% confidence interval (CI). Finally, stepwise linear regression analysis was used to investigate association between persistent neurological impairments and the primary outcome NDI at one- and two-year follow-up. Sex, age, surgery and randomization were also entered into the model, and significant associations were reported with B coefficient, 95% CI and the adjusted coefficient of determination (adj. R2). The required sample size for the study was determined based on the primary outcome of the RCT; NDI with an expected 10% between-group difference, assuming 80% power, and a level of significance of 5%, with allowance for drop-outs (n = 82). A total of 202 patients were recruited. Statistical Package for the Social Sciences (SPSS) Statistics 26 was used for statistical analyses. All analyses were performed by a university statistician and according to intention-to-treat principles, keeping the initial randomization. A p < 0.05 was regarded as significant.

## Results

### Postoperative neurological outcomes: between-group difference and change over time

There was no difference in postoperative neurological outcomes between SPT and SA at follow-up (p > 0.07), except that significantly more patients in the SA group had recovered arm reflexes at one-year follow-up (p = 0.017) (Table 2). The prevalence of neurological impairments in sensibility, motor function and a positive Spurling test decreased significantly over time in both groups (p < 0.04), whereas neurological impairment in arm reflex was significantly decreased at one year only in the SA group (p = 0.04). Post-hoc tests showed that significant changes in neurological outcomes occurred only at one year follow-up and not in the postoperative period from one to two-year follow-up (p > 0.34).

**Table 2:** Prevalence of persistent neurological impairments and neurological recovery at one- and two-year follow-up in participants with cervical radiculopathy who underwent surgery and postoperative rehabilitation.

Postoperative neurological outcomes	One-year follow-up				Two-year follow-up			
	N	Total	SPT	SA	N	Total	SPT	SA
Sensibility								
Impairment prick touch	153	77 (50)	40 (50)	37 (51)	134	72 (54)	39 (52)	33 (56)
Recovery prick touch	151	53 (35)	27 (34)	26 (36)	132	42 (32)	25 (34)	17 (29)
Impairment light touch	152	76 (50)	37 (47)	39 (53)	135	67 (50)	32 (43)	35 (58)
Recovery light touch	150	46 (31)	24 (31)	22 (31)	133	39 (29)	25 (34)	14 (24)
Motor function								
Impairment motor function	153	52 (34)	30 (38)	22 (30)	134	41 (31)	26 (35)	15 (25)
Recovery motor function	151	74 (49)	40 (51)	34 (47)	132	66 (50)	36 (51)	29 (49)
Arm reflexes								
Impairment reflex arm	145	68 (47)	38 (51)	30 (43)	130	67 (52)	38 (53)	29 (50)
Recovery reflex arm	138	41 (30)	15 (21)	26 (39)	125	34 (27)	18 (26)	16 (29)
Spurling test								
Positive Spurling test	118	27 (23)	15 (25)	12 21)	107	40 (37)	22 (38)	18 (37)
Recovery Spurling test	111	49 (44)	25 (44)	24 (44)	98	34 (35)	19 (36)	15 (33)

Results are presented with number (n) and percentage (%) as n (%). Persistent neurological impairments includes patients presenting at least one abnormal response, and neurological recovery includes patients with preoperative impairment presenting normal neurology at follow-up.

### Persistent neurological impairments in relation to NDI score

Persistent neurological impairments were common at follow-up (one year, n = 121, 79%, and two-year, n = 110, 82%), most frequently impairments in sensibility and reflex arm (Table 3). Persistent impairments in sensibility i.e. prick and light touch were strongly associated (p < 0.001); further, impairments in sensibility were associated with impairments in muscle function, and a positive Spurling test (p < 0.001). Impairments in reflex arm were solely associated with impairments in muscle function (p < 0.001) (Table 3). Preoperative impairment in sensitivity assessed with light touch, a positive Spurling test, age and surgical procedure were significant predictors of one-year persistent neurological impairments (p < 0.04) (Table 4). They explained together between 16% (Cox and Snell R square) and 24% (Nagelkerke R squared) of the variance in a persistent positive Spurling test at one year follow-up (p < 0.001) (Table 4). A persistent positive Spurling test (14.3, 95% CI 7.2–21.5, p < 0.001) and neurological impairments in motor function at one year (6.4, 95% CI 0.2–12.6, p = 0.04) (adj. R^2^ = 0.18, p < 0.001), and neurological impairments in prick touch at two-year (15.7, 95% CI 8.9–22.55, p < 0.001) (adj. R^2^ = 0.19, p < 0.001) were significantly associated with higher NDI score.

**Table 3:** Univariate associations between persistent neurological impairments at one year follow-up among participants with cervical radiculopathy who underwent surgery and postoperative rehabilitation.

Persistent neurological impairments	Impairment prick touch	Impairment light touch	Impairment muscle function	Impairment reflex arm	Positive Spurling test
	N = 77 (50%)	N = 76 (50%)	N = 52 (34%)	N = 68 (47%)	N = 27 (23%)
Impairment prick touch	–	N = 152 OR 83.8 (28.8–243.8) P < 0.001	N = 153 OR 3.8 (1.8–7.7) P < 0.001	ns	N = 118 OR 6.4 (2.1–20.0) P = 0.001
Impairment light touch	N = 152 OR 83.8 (28.8–243.8) P < 0.001	–	N = 152 OR 4.4 (2.1–9.2) P < 0.001	ns	N = 117 OR 6.4 (2.0–20.1) P = 0.001
Impairment muscle function	N = 153 OR 3.8 (1.8–7.7) P < 0.001	N = 152 OR 4.4 (2.1–9.2) P < 0.001	–	N = 145 OR 3.4 (1.6–7.0) P = 0.001	ns
Impairment reflex arm	ns	ns	N = 145 OR 3.4 (1.6–7.0) P = 0.001	–	ns
Positive Spurling test	N = 118 OR 6.4 (2.1–20.0) P = 0.001	N = 117 OR 6.4 (2.0–20.1) P = 0.001	ns	ns	–

Results are presented with odds ratio (OR), 95% confidence interval, and p values.

**Table 4:** Preoperative predictors of one-year persistent neurological impairments.

Preoperative factors	One year neurological impairments				
	Neurological impairment prick touch	Neurological impairment light touch	Neurological impairment motor function	Neurological impairment reflex arm	Positive spurling test
N	108	107	108	102	106
Sex	–	–	–	–	–
Age	–	–	–	–	OR 0.89 (0.82–0.95) P = 0.001
Anterior surgery	–	–	OR 3.7 (1.3–10.6) P = 0.016	OR 2.8 (1.1–7.4) P = 0.039	OR 0.19 (0.05–0.66) P = 0.009
Randomization postoperative rehabilitation	–	–	–	–	–
Neurological impairment prick touch	–	–	–	–	–
Neurological impairment light touch	OR 4.5 (1.7–11.6) P = 0.002	OR 3.4 (1.3–8.9) P = 0.010	–	–	–
Neurological impairment motor function	–	–	–	–	–
Neurological impairment reflex arm	–	–	–	–	–
Positive Spurling test	–	–	–	–	OR 3.5 (1.1–10.9) P = 0.031

Results from multiple forward logistic regression analysis are presented with odds ratio (OR), 95% confidence interval, and p values.

## Discussion

The present study is to our knowledge the first RCT study also reporting evaluation of neurological outcomes based on a clinical examination with a two-year follow-up after surgery and postoperative rehabilitation in patients with CR. The results showed no between- group differences, that persistent neurological impairments were common at follow-up, and the outcomes are less positive than previous results from RCTs after surgery reporting above 80% improvements in neurological function^21,22^. The differences may be explained by the fact that both Phillips et al.^21^ and Burkus et al.^22^ only included individuals with single level cervical disc disease, whereas up to three segmental levels were operated on in the present study, although we did not find differences in outcome within the group related to the number of segmental levels. Other differences regarding study population, surgical procedure, symptom duration before surgery and the assessment and interpretation of neurological findings may explain the differences. It is noticeable that Phillips et al.^21^ only reported pooled data of neurology, including no separate reports of sensibility, motor function and reflexes. Neurological maintenance was reported as a successful outcome by both Phillips et al.^21^ and Burkus et al.^22^, while in the present study the primary outcome was persistent neurological impairments to highlight persistent disability with regard to prognosis. However, neurological impairments improved over time in both groups with the exception of arm reflexes, and the results are comparable to the results reported by Ludvigsson et al.^9,10^, who reported improvement in neurological function, except for reflexes after a neck-specific exercise programme in individuals with chronic WAD. A programme including neck-specific exercise has also been reported to reduce self-reported arm pain in CR patients in the present cohort^13^, and is hereby supported in a clinical examination. In a meta-analysis regarding exercise in individuals with CR, Liang et al.^29^ concluded that individually and carefully chosen exercise may improve pain and disability. A possible explanation to the reported effects of neck-specific exercise on pain and disability may be its impact on neck muscle endurance and neck muscle function resulting in improved stabilization of the cervical spine with reduced pressure on the disc and increased intervertebral foramina space, and thereby relieving pressure on the nerve root^30–33^. In the present study, persistent neurological impairments in sensibility and arm reflexes were most frequent at follow-up. Recovery of neurological function was more often observed for preoperative impairments in motor function and a positive Spurling test, whereas, a persistent positive Spurling test and impairments in motor function were associated with poorer outcome for one-year patient-reported neck disability About two thirds of patients who underwent surgery for CR due to cervical disc disease experienced residual pain and disability with reduced work ability^7^. This is a major societal problem, as the mean age of those having surgery is about 45 years. Most studies focus on surgical techniques and not on rehabilitation outcomes including physical function. The main purpose of surgery is to decompress the cervical nerve root to improve neurological function and decrease pain and disability. Assessment of neurological outcomes based on a clinical examination of neurological function with follow-up is important to inform patients of the prognosis, and to identify those with a poor outcome in need of further intervention to optimize neck-specific function. Knowledge about clinical outcomes following postoperative rehabilitation is limited, hence the present study being of clinical importance. No between-group differences in neurological outcomes were reported, and the results correspond with previous results from the RCT^13^. Between-group differences in neurological outcomes were investigated according to the original RCT study design. Ludvigsson et al.^9,10^ reported improvements in radiating pain and neurological deficits after a neck-specific exercise program in patients with chronic whiplash. The significant improvements in neurological outcomes in both groups in the present study are more likely to predominantly result from surgery considering that the patients presented with long-standing pain and specific MRI findings of CR..

### Limitations

This was a secondary analysis of an RCT, thus, the sample-size calculation was based on the main outcome NDI in the RCT. Next, the loss of follow-up (21% at 1 year and 30% at 2 years) was regarded as acceptable given that the follow-up was based on a clinical examination and that some patients had long way to travel to attend follow-up. Moreover, there was no difference at baseline between the patients who were included in the analysis and the patients lost to follow-up, suggesting no impact of loss to follow-upon the results. Patients had about 80% good global outcome, which is comparable with earlier studies^3,6^ that may be interpreted as having a representative study sample. Symptomatic levels for the present study population were predominantly in the lower part of the cervical spine, and symptoms of radiculopathy from the segmental levels above C4 were not evaluated^34^. Another limitation is that assessment of sensibility is rather exhaustive, with overlap between nerve roots^26^. In the present study, sensibility was assessed in each dermatome on both sides for light touch and prick touch, but we only reported the presence of impairment as at least one abnormal response in one dermatome. Despite, overall, being the best possible tests available and commonly performed in clinical examinations^14^, clinical tests should be interpreted with caution^14,15,26,35^. To enhance reliability in the present study, the tests were standardized and performed by a few well-trained, experienced test leaders who were musculoskeletal specialists, and the patient were followed by the same blinded test leader at baseline and follow-ups. Results from a multi-centre study may be more generalizable to health care in general, but they offer less control over the performance of the intended interventions. In the present study, all physiotherapists received standardized written instructions and half a day of practical training, although we had less control over the SA group receiving pragmatic care.

## Conclusion

There was no between-group difference in neurological outcomes between patients undergoing structured postoperative rehabilitation compared with standard approach after surgery for CR. Although, neurological outcomes improved over time, persistent neurological impairments were common at follow-up, most frequently impairments in sensibility and reflex arm. A persistent positive Spurling test, impairments in motor function, and sensitivity were significantly associated with poorer outcome for patient-reported neck disability. The results contribute to inform prognosis regarding recovery of neurological outcomes in patients with CR undergoing surgery and rehabilitation in relation to patient-reported neck-disabilty outcomes. Research should aim to further understand recovery of neurological function to better target interventions to improve clinical outcomes in patients with CR.

## Data Availability

Data can be available from Johanna Wibault upon reasonable request and after ethical permission.
